# Predictive value of respiratory mechanics for the risk of unilateral pulmonary edema following minimally invasive cardiac surgery: An observational study

**DOI:** 10.1097/MD.0000000000041568

**Published:** 2025-02-14

**Authors:** Qiaolian Fan, Feng Chen, Mingguang Chen, Fenghui Lin, Yimin Xue

**Affiliations:** a Fourth Department of Critical Care Medicine, Shengli Clinical Medical College of Fujian Medical University, Fujian Provincial Hospital, Fuzhou University Affiliated Provincial Hospital, Fujian Provincial Key Laboratory of Emergency Medicine, Fuzhou, Fujian, China; b Department of Emergency, Shengli Clinical Medical College of Fujian Medical University, Fujian Provincial Hospital, Fuzhou University Affiliated Provincial Hospital, Fujian Provincial Key Laboratory of Emergency Medicine, Fuzhou, Fujian, China.

**Keywords:** airway resistance, minimally invasive cardiac surgery, static lung compliance, unilateral pulmonary edema

## Abstract

This study aimed to investigate the predictive effect of static lung compliance (SLC) and airway resistance (AR) in patients undergoing minimally invasive cardiac surgery (MICS) with unilateral pulmonary edema (UPE). A total of 245 patients who underwent MICS via cardiopulmonary bypass and minimal right lateral thoracic incision port access were enrolled, with immediate postoperative SLC and AR data collected upon intensive care unit (ICU) admission. Cutoff values for grouping patients into high (H-) and low (L-) compliance/resistance categories were determined using receiver-operating characteristic curves and Youden indexes. The primary outcome was the incidence of radiographically and clinically defined UPE within the first 24 hours postoperatively, while the secondary outcomes included duration of mechanical ventilation, length of ICU stay, total hospitalization days, in-hospital mortality, and the highest sequential organ failure assessment (SOFA) and acute physiology and chronic health evaluation II (APACHE-II) scores within the first 24 hours post-surgery. Results showed that patients in the L-compliance group (SLC < 40 mL/cmH2O) had longer durations of mechanical ventilation, length of ICU stay, and total hospitalization days, along with higher SOFA and APACHE-II scores compared to those in the H-compliance group (SLC ≥ 40 mL/cmH2O) (*P* < .05), although there was no significant difference in in-hospital mortality. Conversely, patients in the H-resistance group (AR ≥ 11 cm H_2_O/[L·s]) exhibited longer durations of mechanical ventilation, length of ICU stay, and total hospitalization days, as well as significantly higher SOFA, APACHE-II scores, but lower in-hospital mortality rates than those in the L-resistance group (AR < 11 cm H_2_O/[L·s]) (*P* < .05). In summary, immediate postoperative SLC < 40 mL/cm H_2_O and AR > 11 cm H_2_O/(L·s) are potentially valuable indicators for predicting postoperative UPE in patients undergoing MICS.

## 1. Introduction

In recent years, advancements in cardiac surgery and extracorporeal circulation technology have led to the rapid development of minimally invasive cardiac surgery (MICS), particularly total thoracoscopic cardiac surgery with cardiopulmonary bypass (CPB).^[1]^ Compared to conventional open thoracotomies, video-assisted thoracic surgery offers minimal invasiveness, improved recovery, reduced hospital stays, and better aesthetic outcomes.^[2]^ Thus, the acceptance and prevalence of minimally invasive surgeries are growing. Unilateral pulmonary edema (UPE) is 1 of the most serious pulmonary complications of MICS, with variable clinical impact and severity, often resulting in significant perioperative mortality. Reports indicate that 0.6% to 25% of patients experience this complication.^[3–6]^ Current understanding suggests that the pathogenesis of this complication is influenced by increased lung capillary permeability and elevated pulmonary capillary pressure.^[7–9]^ When lung capillary permeability increases, fluid from pulmonary edema enters the alveoli, resulting in hypoxemia and decreased lung compliance. Therefore, patients with perioperative respiratory failure depend on mechanical ventilation for survival. Given the importance of lung-protective ventilation, respiratory mechanics is an essential aspect for the clinical management of patients with UPE following MICS. In addition to quantifying the mechanical parameters of the respiratory system, it can also facilitate the assessment of disease progression and the modification of ventilation strategies from an alternative perspective.

Previous studies suggest that basic indicators, such as airway driving pressure, are strong independent predictors of mortality in acute respiratory distress syndrome (ARDS).^[10]^ Another study revealed that static lung compliance (SLC) may be an early predictor of intact neurologic survival in ARDS patients after cardiac arrest.^[11]^ However, few studies have been conducted on the role of respiratory mechanics parameters as an aid to mechanical ventilation in the presence of UPE following MICS. The objective of this study was to identify the optimal cutoff values for SLC and airway resistance (AR) in predicting the diagnosis of UPE and to assess their efficacy in predicting the outcomes of MICS patients.

## 2. Methods

### 2.1. General data

The study enrolled patients who underwent MICS with CPB and port access through a minimal right lateral thoracic incision from March 1, 2020, to March 1, 2023. In this study, we extracted the electronic medical records with the operation title code of MICS in the operative records. All data were obtained from the electronic medical record system and the data were accessed from January 1, 2024, to January 31, 2024. The inclusion criteria were as follows: patients who underwent total thoracoscopic cardiac surgery with CPB and 1-lung ventilation technique; male or female, aged 18 to 80 years; body mass index of 18 to 25 kg/m^2^; all patients were transferred to the cardiac intensive care unit (ICU) and received invasive mechanical ventilation postoperatively, immediate postoperative SLC and AR data could be obtained after ICU admission. Exclusion criteria were post-surgery hemorrhagic shock, cardiac rupture, and cardiac tamponade; perioperative use of intra-aortic balloon pump and extracorporeal membrane oxygenation; severe pulmonary dysfunction; inability to receive adequate sedative and analgesic treatment. This study protocol adhered to the principles outlined in the Declaration of Helsinki (2013 revision). This retrospective observational study was reviewed and approved by the ethics committee of Fujian Provincial Hospital (permit no. K2024-01-008).

### 2.2. Respiratory mechanics measurements

According to the gas equation of motion,^[12]^ respiratory work primarily overcomes the sum of work performed by AR, elastic resistance, and positive end-expiratory pressure (PEEP) in mechanically ventilated patients, as follows:


$$\begin{array}{l} P_{rs}=P_{ao}+P_{mus}=Flow\times R+V/C+K \end{array}$$

Where *P*_rs_ is the total respiratory system pressure, *P*_ao_ is the airway opening pressure, *P*_mus_ is the pressure generated by the patient’s respiratory muscles, Flow is the inspiratory flow rate, and *R* is the inspiratory resistance. This product represents the pressure required to overcome frictional forces generated by flow through the endotracheal tube and airways (also called resistive pressure). *V* is the lung volume above functional residual capacity, *C* is the static compliance of the respiratory system, and *V*/*C* is the pressure necessary to overcome respiratory elasticity. *K* represents the basal pressure, which is the sum of extrinsic and intrinsic PEEP.

Immediately following MICS, all patients were transferred to the ICU. Patients who had not resumed spontaneous breathing (*P*_mus_ = 0) due to anesthesia were subjected to passive ventilation. In the volume-controlled ventilation with square wave mode, the SLC and AR were obtained by the occlusion method by using the inspiratory and expiratory hold functions of the ventilator (Puritan-Bennett 840; Covidien, Boulder) (Flow = 0), as follows:


$$\begin{array}{l} C=V/(P_{plat}-K) \end{array}$$

$$\begin{array}{l} R=(P_{peak}-P_{plat})/Flow \end{array}$$

Where *P*_peak_ is peak airway pressure, *P*_plat_ is end-inspiratory plateau pressure. The respiratory mechanics data was averaged across 3 measurements.

Based on the principles above, we performed Puritan-Bennett PB840 ventilators equipped with respiratory mechanics function to obtain immediate postoperative SLC and AR data after ICU admission. All patients were ventilated using Puritan-Bennett PB840 ventilators (Covidien, Boulder) in volume control mode (a tidal volume of 8 mL/kg of ideal body weight, 0.4 of FiO_2_, respiratory rate of 12 breaths/minute, inspiratory-to-expiratory ratio of 1:2, inspiratory time of 1.0 seconds, PEEP of 5 cmH_2_O, flow velocity waveform of a square wave, inspiratory breath-hold of 3–4 seconds) immediately after surgery. The SLC and AR displayed by the ventilator were recorded. The respiratory mechanics data were averaged across 3 measurements. Additionally, all ventilator parameters were recorded in the electronic medical record by experienced ICU nurses. Following the measurement, the ventilator parameters were modified in accordance with the patient’s clinical status.

### 2.3. Observed indicators

**Figure 1.**
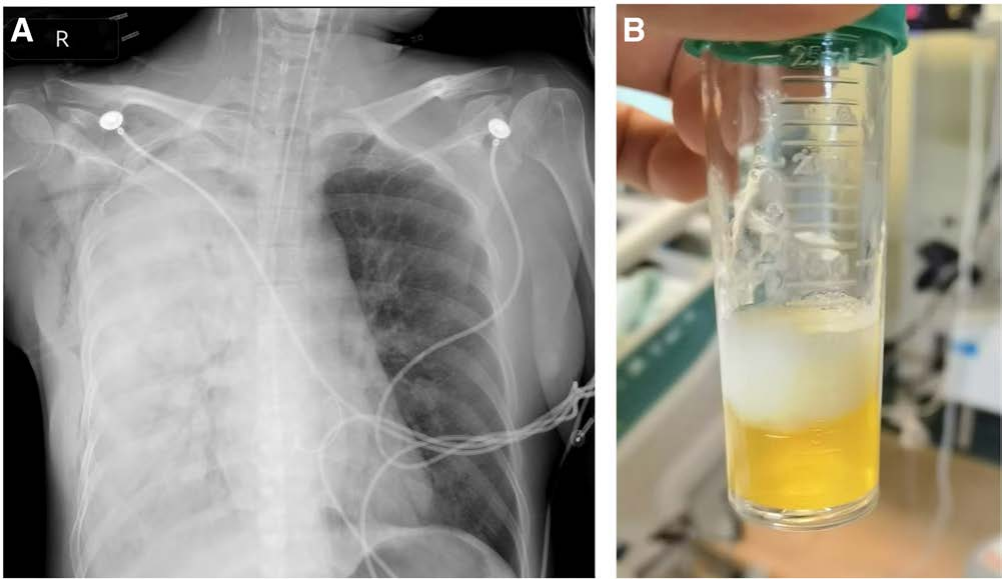
(A) Bedside chest radiograph showed significant lung consolidation on the right side corresponding to the surgical approach, but the left side was relatively normal. (B) Postoperative bronchoscopy showed a large amount of yellowish airway secretions from the right bronchus, which was removed by negative pressure suction.

The primary outcome was the occurrence of radiographically and clinically defined UPE within the first 24 hours post-surgery. All enrolled patients had a chest x-ray taken at the bedside. A newly developed UPE refers to evident imaging findings (over 20% opacification of the right hemithorax, Fig. 1A) and abundant transparent yellowish airway secretions (Fig. 1B).^[5,13]^ The secondary outcomes included several critical measures: the duration of mechanical ventilation, length of ICU stay, total hospitalization days, and in-hospital mortality. Additionally, the highest sequential organ failure assessment (SOFA) score and the highest acute physiology and chronic health evaluation II (APACHE-II) score within the first 24 hours post-surgery were also evaluated.^[14,15]^

### 2.4. Statistical analysis

The statistical analysis of this study was conducted with IBM SPSS Statistics version 25.0 (Chicago) and GraphPad Prism v.9.0 software (GraphPad Inc., La Jolla). The threshold for statistical significance was set at *P* < .05 for all analyses, which were 2-tailed. For data with a normal distribution, measurement variables were shown as the mean ± standard deviation. In contrast, the measurement variables with non-normal distribution were represented by the median (*P50*) and interquartile range (IQR) (25%–75%). Normally distributed data were analyzed using the *T*-test, while non-normally distributed data were evaluated using the rank-sum test (Mann–Whitney *U* test or Kruskal–Wallis H rank-sum test). Additionally, categorical variables were expressed as frequency (n) and percentage (%) and compared using the χ^2^ or Fisher exact test. The sensitivity, specificity, and cutoff values for SLC and AR were determined in accordance with the receiver-operating characteristic (ROC) curve and Youden Index calculation. According to the cutoff values, patients were divided into 2 groups. For linear regression model or logistics regression model, *b* (regression coefficient), odds ratio (OR), the 95% confidence interval (95%CI), and *P* values were reported.

## 3. Results

### 3.1. Clinical characteristics

**Figure 2.**
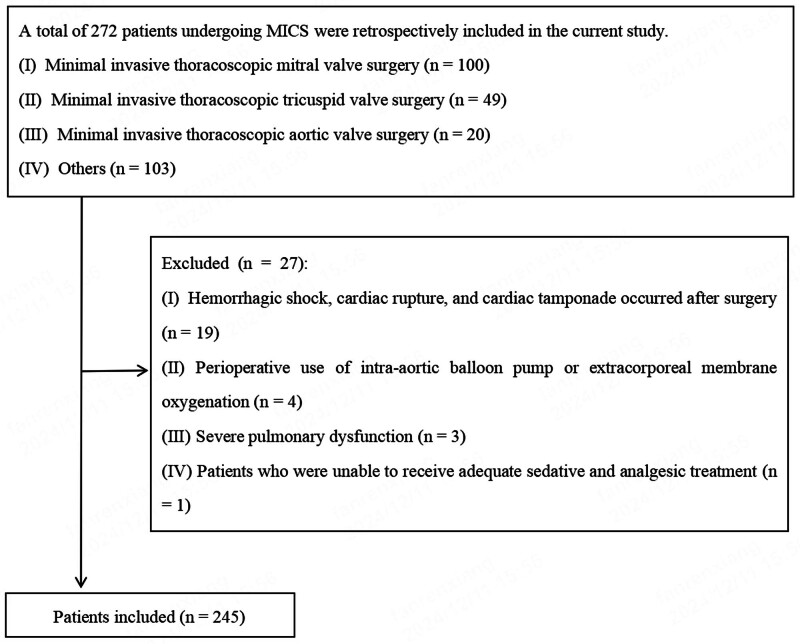
The data selection procedures and standards.

In this study, 272 patients admitted to the ICU after MICS were enrolled retrospectively. Figure 2 shows the data selection procedures and standards, while Table 1 presents the basic clinical characteristics of the populations.

**Table 1:** Comparison of baseline clinical characteristics between UPE and non-UPE patients.

Characteristics	Overall (n = 245)	UPE (n = 31)	Non-UPE (n = 214)	*P*
Age, median (IQR)	52.0 (42.5, 59.0)	56.0 (48.0, 61.0)	51.0 (41.0, 59.0)	.057
Females gender, n (%)	133.0 (54.3)	16.0 (51.6)	117.0 (54.7)	.749
BMI, median (IQR) (kg/m^2^)	22.1 (21.2, 22.9)	22.4 (20.6, 23.8)	22.0 (21.2, 22.9)	.173
Smoking history, n (%)	40.0 (16.3)	8.0 (25.8)	32.0 (15.0)	.127
Hypertension, n (%)	46.0 (18.8)	7.0 (22.6)	39.0 (18.2)	.562
Diabetes, n (%)	13.0 (5.3)	3.0 (9.7)	10.0 (4.7)	.463
Atrial fibrillation, n (%)	42.0 (17.1)	13.0 (41.9)	29.0 (13.6)	<.001
NYHA functional class, n (%)				
I to II	107.0 (43.7)	7.0 (22.6)	100.0 (46.7)	.011
III to IV	138.0 (56.3)	24.0 (77.4)	114.0 (53.3)	
Operative time, median (IQR) (min)	301.0 (250.0, 370.0)	390.0 (320.0, 440.0)	290.0 (240.0, 360.0)	<.001
CPB time, median (IQR) (min)	163.0 (111.0, 214.0)	232.0 (203.0, 295.0)	150.5 (102.8, 203.0)	<.001
ACCT, median (IQR) (min)	103.0 (55.5, 141.0)	144.0 (127.0, 178.0)	95.0 (52.8, 135.5)	<.001

ACCT = aortic cross-clamp time, BMI = body mass index, CPB = cardiopulmonary bypass, IQR = interquartile range, NYHA = New York Heart Association, UPE = unilateral pulmonary edema.

**Figure 3.**
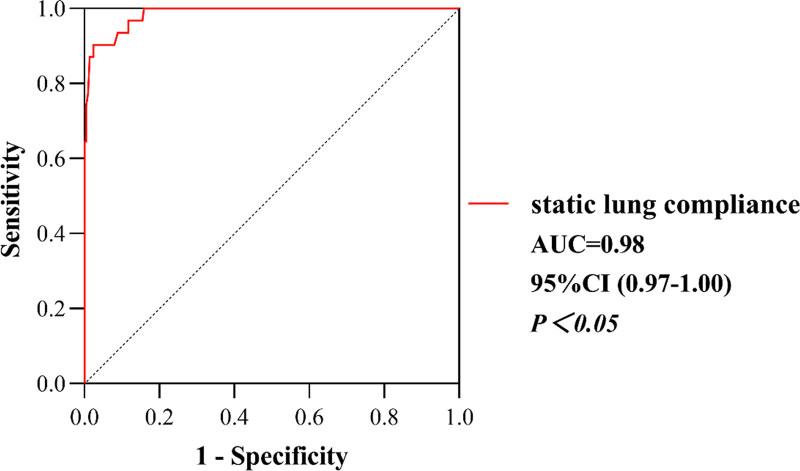
The ROC curve for SLC to predict postoperative UPE. ROC = receiver-operating characteristic, SLC = static lung compliance, UPE = unilateral pulmonary edema.

**Figure 4.**
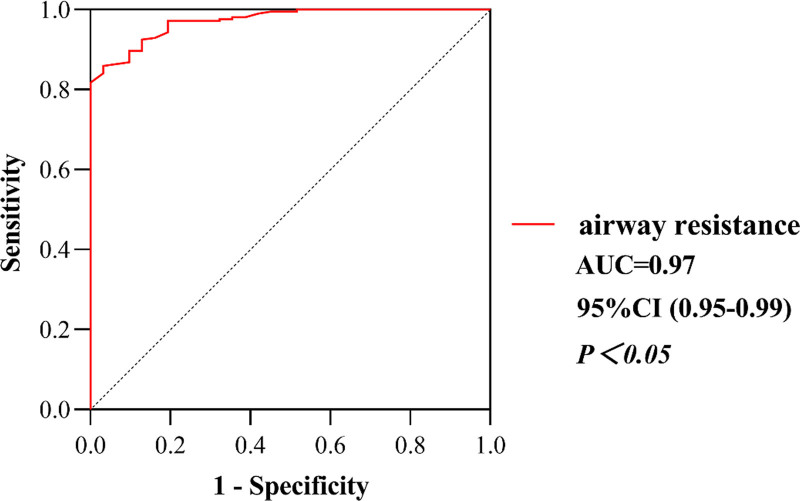
The ROC curve for AR to predict postoperative UPE. AR = airway resistance, ROC = receiver-operating characteristic, UPE = unilateral pulmonary edema.

For SLC, the ROC curve indicated a cutoff value of 40 mL/cmH_2_O, with a specificity of 97.7% and a sensitivity of 90.3%, while the critical value of AR was 11 cm H_2_O/(L·s), with a specificity of 86% and a sensitivity of 96.8%. With postoperative UPE, the area under the curve for SLC and AR was 0.98 (*P *< .05, Fig. 3) and 0.97 (*P* < .05, Fig. 4), respectively. According to the cutoff value, the patients were divided into 2 groups: the high SLC group (H-compliance group, SLC ≥ 40 mL/cmH_2_O, n = 212) and the low SLC group (L-compliance group, SLC < 40 mL/cmH_2_O, n = 33). Similarly, patients were divided into the high AR group (H-resistance group, AR ≥ 11 cmH_2_O/(L·s), n = 60) and the low AR group (L-resistance group, AR < 11 cmH_2_O/(L·s), n = 185).

### 3.2. Correlation between SLC and postoperative outcomes

General comparative data for all patients included in the study are reported in Table 2 and further explored in Tables 3 and 4. In comparing SLC groups, the duration of mechanical ventilation, length of ICU stay, and total hospitalization days were statistically significant. In a multivariate linear regression model and another multivariate logistic regression model adjusting for atrial fibrillation, cardiac function, operative time, CPB time, ACCT, the duration of mechanical ventilation (*b *= 3.919, 95% CI = 3.256–4.581, *P < *.001), length of ICU stay (*b *= 4.281, 95% CI = 3.561–5.002, *P < *.001), and total hospitalization days (*b* = 8.121, 95% CI = 4.470–11.771, *P < *.001) were all extended in the L-compliance group than in the H-compliance group. Additionally, the SOFA (*b* = 2.983, 95% CI = 2.229–3.737, *P < *.001) and APACHE-II scores (*b *= 6.563, 95% CI = 4.940–8.816, *P < *.001) were higher in the L-compliance group than in the H-compliance group. However, there were no significant associations between the 2 groups regarding the incidence of in-hospital mortality (OR = 0.155, 95% CI = 0.022–1.081, *P* = .06).

**Table 2:** Basic characteristics of the H-compliance group and the L-compliance group.

Characteristics	Overall (n = 245)	L-compliance group (n = 185)	H-compliance group (n = 60)	*P*
Age, median (IQR)	52.0 (42.5, 59.0)	54.0 (47.5, 59.0)	51.0 (41.0, 59.0)	.231
Females gender, n (%)	133.0 (54.3)	16.0 (48.5)	117.0 (55.2)	.472
BMI, median (IQR) (kg/m^2^)	22.1 (21.2, 22.9)	22.4 (20.6, 23.6)	22.0 (21.2, 22.9)	.282
Smoking history, n (%)	40.0 (16.3)	7.0 (21.2)	33.0 (15.6)	.414
Hypertension, n (%)	46.0 (18.8)	6.0 (18.2)	40.0 (18.9)	.925
Diabetes, n (%)	13.0 (5.3)	2.0 (6.1)	11.0 (5.2)	1.000
Atrial fibrillation, n (%)	42.0 (17.1)	12.0 (36.4)	30.0 (14.2)	.002
NYHA functional class, n (%)				
I to II	107.0 (43.7)	7.0 (21.2)	100.0 (47.2)	.005
III to IV	138.0 (56.3)	26.0 (78.8)	112.0 (52.8)	
Operative time, median (IQR) (min)	301.0 (250.0, 370.0)	380.0 (320.0, 437.5)	290.0 (240.0, 360.0)	<.001
CPB time, median (IQR) (min)	163.0 (111.0, 214.0)	224.0 (185.0, 292.5)	151.5 (102.2, 203.0)	<.001
ACCT, median (IQR) (min)	103.0 (55.5, 141.0)	138.0 (107.5, 173.0)	95.5 (52.2, 138.7)	<.001

ACCT = aortic cross-clamp time, BMI = body mass index, CPB = cardiopulmonary bypass, IQR = interquartile range, NYHA = New York Heart Association.

**Table 3:** The linear regression of SLC.

Outcomes	Non-adjusted model			Adjusted model		
	*b*	95% CI	*P*	*b*	95% CI	*P*
Duration of mechanical ventilation	4.260	3.646 to 4.878	<.001	3.919	3.256 to 4.581	<.001
Length of ICU stay	4.814	4.137 to 5.490	<.001	4.281	3.561 to 5.002	<.001
Total hospitalization d	11.345	7.899 to 14.790	<.001	8.121	4.470 to 11.771	<.001
SOFA score	4.326	3.500 to 5.153	<.001	2.983	2.229 to 3.737	<.001
APACHE-II score	8.184	7.122 to 10.446	<.001	6.563	4.490 to 8.186	<.001

APACHE-II = acute physiology and chronic health evaluation II, ICU = intensive care unit, SLC = static lung compliance, SOFA = sequential organ failure assessment.

**Table 4:** The logistics regression of SLC.

Outcomes	Non-adjusted model			Adjusted model		
	OR	95% CI	*P*	OR	95% CI	*P*
In-hospital mortality	14.483	2.539 to 82.614	.003	0.155	0.022 to 1.081	.06

SLC = static lung compliance.

### 3.3. Correlation between AR and postoperative outcomes

The primary general data are summarized in Table 5 and deeply analyzed in Tables 6 and 7. Based on the AR, statistical differences existed between groups. Adjusting for age, atrial fibrillation, operative time, CPB time, ACCT, the regression equation showed that the H-resistance group had longer duration of mechanical ventilation (*b *= 2.404, 95% CI = 1.787–3.021, *P < *.001), length of ICU stay (*b *= 2.629, 95% CI = 1.960–3.299, *P < *.001), and total hospitalization days (*b *= 6.958, 95% CI = 3.944–9.972, *P < *.001) compared to the L-resistance group. In addition, the H-resistance group demonstrated an increase in the SOFA score (*b *= 2.038, 95% CI = 1.390–2.686, *P < *.001), APACHE-II score (*b *= 3.445, 95% CI = 2.113–4.777, *P < *.001), but showed a decrease in in-hospital mortality (OR = 0.059, 95% CI = 0.005–0.626, *P *= .019) compared to the L-resistance group.

**Table 5:** Basic characteristics of the H-resistance group and the L-resistance group.

Characteristics	Overall (n = 245)	L-resistance group (n = 185)	H-resistance group (n = 60)	*P*
Age, median (IQR)	52.0 (42.5, 59.0)	51.0 (40.5, 59.0)	56.0 (46.2, 60.0)	.035
Females gender, n (%)	133.0 (54.3)	98.0 (53.0)	35.0 (58.3)	.469
BMI, median (IQR) (kg/m^2^)	22.1 (21.2, 22.9)	22.0 (21.2, 22.9)	22.3 (20.8, 23.3)	.361
Smoking history, n (%)	40.0 (16.3)	29.0 (15.7)	11.0 (18.3)	.628
Hypertension, n (%)	46.0 (18.8)	31.0 (16.8)	15.0 (25.0)	.155
Diabetes, n (%)	13.0 (5.3)	10.0 (5.4)	3.0 (5.0)	1.000
Atrial fibrillation, n (%)	42.0 (17.1)	26.0 (14.1)	16.0 (26.7)	.024
NYHA functional class, n (%)				
I to II	107.0 (43.7)	85.0 (45.9)	100.0 (54.1)	.137
III to IV	138.0 (56.3)	21.0 (35.0)	39.0 (65.0)	
Operative time, median (IQR) (min)	301.0 (250.0, 370.0)	280.0 (230.0, 340.0)	380.0 (322.5, 431.2)	<.001
CPB time, median (IQR) (min)	163.0 (111.0, 214.0)	140.0 (98.0, 197.0)	216.0 (183.5, 265.8)	<.001
ACCT, median (IQR) (min)	103.0 (55.5, 141.0)	85.0 (49.0, 128.5)	141.0 (114.5, 168.5)	<.001

ACCT = aortic cross-clamp time, BMI = body mass index, CPB = cardiopulmonary bypass, IQR = interquartile range, NYHA = New York Heart Association.

**Table 6:** The linear regression of AR.

Outcomes	Non-adjusted model			Adjusted model		
	*b*	95% CI	*P*	*b*	95% CI	*P*
Duration of mechanical ventilation	2.611	2.053 to 3.169	<.001	2.404	1.787 to 3.021	<.001
Length of ICU stay	2.997	2.381 to 3.612	<.001	2.629	1.960 to 3.299	<.001
Total hospitalization days	9.650	6.949 to 12.350	<.001	6.958	3.944 to 9.972	<.001
SOFA score	3.349	2.685 to 4.012	<.001	2.038	1.390 to 2.686	<.001
APACHE-II score	5.814	4.408 to 7.220	<.001	3.445	2.113 to 4.777	<.001

APACHE-II = acute physiology and chronic health evaluation II, AR = airway resistance, ICU = intensive care unit, SOFA = sequential organ failure assessment.

**Table 7:** The logistics regression of AR.

Outcomes	Non-adjusted model			Adjusted model		
	OR	95% CI	*P*	OR	95% CI	*P*
In-hospital mortality	16.727	1.914 to 146.218	.011	0.059	0.005 to 0.625	.019

AR = airway resistance, CI = confidence interval, OR = odds ratio.

## 4. Discussion

This retrospective study demonstrated that immediate postoperative SLC decreased to 40 mL/cmH_2_O, while AR increased to 11 cmH_2_O/(L·s) after ICU admission, indicating an increased risk of UPE. Exceeding the warning values for SLC and AR was associated with increased mechanical ventilation duration, prolonged ICU stay and total hospitalization days, as well as higher SOFA and APACHE-II scores. Moreover, in-hospital mortality was increased in the low AR group but not in the low SLC group.

Consistently, port-accessed cardiac surgery using a thoracoscope has assumed a central role in MICS with CPB and 1-lung ventilation technique. Since the advent of MICS, UPE has been regarded as a unique complication for many years, with varying incidences.^[3–6]^ However, the etiology and pathogenesis of UPE are not yet completely understood. It is generally believed that increased lung capillary permeability and elevated pulmonary capillary pressure contribute to the pathogenesis of this disease as a result of lung ischemic-reperfusion injury, impaired lung mechanical barrier, extracorporeal circulation, and inflammatory responses.^[4,7–9,16–18]^ These variables may interact or exert differing degrees of influence on SLC. Based on the above significant factors, the severity of UPE within 24 hours postoperatively ranges from being clinically asymptomatic, a solely radiographic finding, to acute respiratory failure followed by severe life-threatening hypoxemia.^[5,19–21]^ Therefore, UPE has a significant impact on the management of the postoperative respiratory system. To improve patient outcomes, early postoperative monitoring of respiratory mechanics is essential. SLC is the elasticity of lung tissue measured when airflow is temporarily blocked during the respiratory cycle, which is related to the recovery of patients with mechanical ventilation.^[22]^ Normal SLC ranges from 60 to 80 mL/cmH_2_O in ventilated patients.^[12]^ Although its primary role is to reflect the elasticity of lung tissue, the association between quantification of SLC and survival is significant. A previous study showed that patients with COVID-19-associated ARDS who have reduced respiratory system compliance have high mortality rates.^[23]^ Other patient populations, including critically ill patients with pulmonary edema, pneumonia, pulmonary fibrosis, neuromuscular diseases, obesity, and abdominal distension, were also effectively evaluated using SLC.^[24,25]^ Recent literature indicates that in mechanically ventilated patients, SLC may be a promising predictor for extubation failure in patients with acute respiratory failure. The study by Abplanalp et al found that a SLC of <50 mL/cmH_2_O was not only associated with extubation failure but also with an increased inpatient mortality of 11.9% compared to 6.1%.^[26]^ In this study, we selected patients who had undergone MICS with extracorporeal circulation and 1-lung ventilation technique to test the predictive effect of SLC for short-term outcomes. Notably, the results demonstrate a remarkable performance of SLC, significantly exceeding the cutoff value in previous research. In the context of MICS, the SLC warning value can serve as a valuable new observation index for evaluating the risk of UPE.

AR refers to the friction between the tracheal wall and gas molecules, as well as between the gas molecules themselves.^[12]^ An increased AR usually indicates a kinked or obstructed endotracheal tube, intraluminal mucus, or bronchospasm. The literature suggests that, among all the measurements of lung mechanics, AR may be the most closely related to lung structure.^[27]^ Due to the 1-lung ventilation technique, pulmonary contusion may also cause increased AR from lung compression and the pulling operation.^[4]^ Most patients are intubated with a double-lumen endotracheal tube intraoperatively and replaced with a single-lumen endotracheal tube postoperatively. Respiratory secretions can flood the unilateral bronchus and gradually progress to the opposite lung, causing double pulmonary edema.^[5]^ This is also the reason for increased AR. Based on a meta-analysis of mechanically ventilated patients undergoing general anesthesia, AR has been implicated in postoperative pulmonary complications.^[28]^ In addition to the study by Fuller et al, which included more than 1705 mechanically ventilated patients, it has been demonstrated that respiratory mechanics, including AR, driving pressure, and plateau pressure, are risk factors for mortality and ARDS.^[29]^ The results mentioned above were validated in diverse populations. To the best of our knowledge, normal AR on a mechanical ventilator is < 15 cmH_2_O/(L·s).^[12]^ In our study, AR was not significantly greater than normal reference values. In contrast, these changes were more noticeable in the SLC. In models of lung ventilation, the effects of lung tissue compliance generally dominate the mechanics compared to AR, except in cases of airway narrowing or blockage.^[30]^ The SLC may serve as a better clinical predictor for postoperative UPE after MICS. Additionally, the decrease in in-hospital mortality observed in the H-resistance group might be attributed to 2 potential factors: selection bias, and the possibility that patients in this group received more aggressive and tailored treatment protocols. The latter may stem from heightened attention and more intensive management by the medical team, resulting in superior care for these patients.

Considering the complexity of disease progression, it is challenging to identify a definitive indicator that can comprehensively predict risk. The strength of this study lies in its enhancement of methods for the early identification of UPE patients following MICS, which could serve as decision support for clinical intervention. As MICS has become more prevalent, UPE has received increased attention. Previous literature has shown that typical ARDS is associated with decreased lung compliance, which is managed with lung-protective ventilation strategies.^[31]^ Consequently, it is essential to investigate novel and personalized lung protection strategies, as they can potentially enhance overall outcomes. However, the literature examining the role of respiratory mechanics in MICS is limited. Previous lung-protective ventilation strategies did not elaborate on this subject.^[32]^

## 5. Limitations

Even though this study explored the value of respiratory mechanics parameters in predicting the risk of UPE after MICS, several limitations still exist in the study design and implementation. First, it is a single-center retrospective analysis, which may encompass information bias. Additionally, the technical proficiency of MICS varies across different regions and medical settings, which may result in different respiratory mechanics parameters and incidences of UPE postoperatively. Second, the sample size in this study was relatively small, with only 245 patients who underwent MICS included. Moreover, this study focused on lung mechanical changes in the early postoperative period, but we believe that the changes in SLC and AR depend on the patient’s condition. Thus, it is necessary to explore further the changes that occur throughout the various phases of respiratory mechanics after MICS. Finally, respiratory mechanics parameters were obtained using mechanical ventilation monitoring systems, which are widely used in clinical settings but may still introduce measurement errors. Additionally, manually recorded data may be influenced by operator subjectivity. Future studies could consider using more advanced monitoring technologies and automated data collection systems to reduce measurement errors and improve data quality. It is noteworthy to mention that our findings in the present study have the potential to provide clinicians with a cautionary measure of SLC and AR values for better lung-protective ventilation. Therefore, future prospective studies with more centers and larger samples are needed.

## 6. Conclusions

In conclusion, this study demonstrates that immediate postoperative SLC < 40 mL/cmH_2_O and AR > 11 cmH_2_O/(L·s) are potentially valuable indicators for predicting postoperative UPE in patients undergoing MICS. These respiratory mechanics parameters provide critical insights into the early identification of patients at risk for developing UPE, thereby facilitating timely intervention strategies to mitigate adverse outcomes.

## Acknowledgments

We thank the Home for Researchers editorial team (www.home-for-researchers.com) for the language editing service.

## Author contributions

**Conceptualization:** Qiaolian Fan, Yimin Xue.

**Data curation:** Qiaolian Fan, Yimin Xue.

**Formal analysis:** Qiaolian Fan, Mingguang Chen.

**Funding acquisition:** Yimin Xue.

**Investigation:** Qiaolian Fan, Mingguang Chen, Fenghui Lin.

**Methodology:** Qiaolian Fan, Feng Chen, Mingguang Chen, Yimin Xue.

**Project administration:** Feng Chen.

**Supervision:** Feng Chen, Fenghui Lin, Yimin Xue.

**Writing – original draft:** Qiaolian Fan.

**Writing – review & editing:** Yimin Xue.

## References

[1] Yan LL Tang MR Dai XF Chen L-W Fang G-H. Impact of minimally invasive mitral valve surgery on sexual dysfunction in male patients. J Cardiothorac Surg. 2022;17:77. 35421997 10.1186/s13019-022-01814-w PMC9008948

[2] Koichi Y Ise H Ohira S. Manual repositioning of lung hernia after minimally invasive cardiac surgery. J Surg Case Rep. 2019;2019:rjz056. 30886694 10.1093/jscr/rjz056 PMC6413369

[3] Keyl C Staier K Pingpoh C. Unilateral pulmonary oedema after minimally invasive cardiac surgery via right anterolateral minithoracotomy. Eur J Cardiothorac Surg. 2015;47:1097–102. 25123672 10.1093/ejcts/ezu312

[4] Yamashiro S Arakaki R Kise Y Kuniyoshi Y. Prevention of pulmonary edema after minimally invasive cardiac surgery with mini-thoracotomy using neutrophil elastase inhibitor. Ann Thorac Cardiovasc Surg. 2018;24:32–9. 29118307 10.5761/atcs.oa.17-00102 PMC5833138

[5] Jung EY Kang HJ Min HK. Unilateral pulmonary edema after minimally invasive cardiac surgery: a case report. J Chest Surg. 2022;55:98–100. 35115428 10.5090/jcs.21.098 PMC8824640

[6] Tamura T Ito T Yokota S. Incidence of reexpansion pulmonary edema in minimally invasive cardiac surgery. Nagoya J Med Sci. 2019;81:647–54. 31849382 10.18999/nagjms.81.4.647 PMC6892674

[7] Rodrigues AL Lopes CE Romaneli MT Fraga AMA Pereira RM Tresoldi AT. Reexpansion pulmonary edema in children. Rev Paul Pediatr. 2013;31:411–5. 24142327 10.1590/S0103-05822013000300021 PMC4182965

[8] den Hengst WA Gielis JF Lin JY Van Schil PE De Windt LJ Moens AL. Lung ischemia-reperfusion injury: a molecular and clinical view on a complex pathophysiological process. Am J Physiol Heart Circ Physiol. 2010;299:H1283–99. 20833966 10.1152/ajpheart.00251.2010

[9] Sohara Y. Reexpansion pulmonary edema. Ann Thorac Cardiovasc Surg. 2008;14:205–9. 18818568

[10] Amato MB Meade MO Slutsky AS. Driving pressure and survival in the acute respiratory distress syndrome. N Engl J Med. 2015;372:747–55. 25693014 10.1056/NEJMsa1410639

[11] Kim JS Kim YJ Kim M. Impact of lung compliance on neurological outcome in patients with acute respiratory distress syndrome following out-of-hospital cardiac arrest. J Clin Med. 2020;9:527. 32075160 10.3390/jcm9020527 PMC7073731

[12] Walter JM Corbridge TC Singer BD. Invasive mechanical ventilation. South Med J. 2018;111:746–53. 30512128 10.14423/SMJ.0000000000000905 PMC6284234

[13] Khalil NH Anders R Forner AF Gutberlet M Ender J. Radiological incidence of unilateral pulmonary edema after minimally invasive cardiac surgery. J Cardiothorac Vasc Anesth. 2020;34:151–6. 31405722 10.1053/j.jvca.2019.07.006

[14] Matsuda J Kato S Yano H. The Sequential Organ Failure Assessment (SOFA) score predicts mortality and neurological outcome in patients with post-cardiac arrest syndrome. J Cardiol. 2020;76:295–302. 32305260 10.1016/j.jjcc.2020.03.007

[15] Ling L Li Y Li H Li W Zhang H-B. MMP-2 and MMP-9 gene polymorphisms act as biological indicators for ulinastatin efficacy in patients with severe acute pancreatitis. Medicine (Baltimore). 2019;98:e15831. 31192912 10.1097/MD.0000000000015831 PMC6587626

[16] Rebetz J Semple JW Kapur R. The pathogenic involvement of neutrophils in acute respiratory distress syndrome and transfusion-related acute lung injury. Transfus Med Hemother. 2018;45:290–8. 30498407 10.1159/000492950 PMC6257140

[17] Puehler T Friedrich C Lutter G. Outcome of unilateral pulmonary edema after minimal-invasive mitral valve surgery: 10-year follow-up. J Clin Med. 2021;10:2411. 34072399 10.3390/jcm10112411 PMC8198899

[18] Meeker JW Jaeger AL Tillis WP. An uncommon complication of a common clinical scenario: exploring reexpansion pulmonary edema with a case report and literature review. J Community Hosp Intern Med Perspect. 2016;6:32257. 27406463 10.3402/jchimp.v6.32257 PMC4942514

[19] Viox D Dhawan R Balkhy HH. Unilateral pulmonary edema after robotically assisted mitral valve repair requiring veno-venous extracorporeal membrane oxygenation. J Cardiothorac Vasc Anesth. 2022;36:321–31. 33975792 10.1053/j.jvca.2021.03.051

[20] Arima T Tatebayashi T Noji S. Management of fulminating non-cardiogenic pulmonary edema following cardiac surgery. J Surg Case Rep. 2023;2023:rjac625. 36636649 10.1093/jscr/rjac625 PMC9831641

[21] Madershahian N Wippermann J Sindhu D Wahlers T. Unilateral re-expansion pulmonary edema: a rare complication following one-lung ventilation for minimal invasive mitral valve reconstruction. J Card Surg. 2009;24:693–4. 19549047 10.1111/j.1540-8191.2009.00813.x

[22] Li Bassi G Suen JY Dalton HJ.; COVID-19 Critical Care Consortium. An appraisal of respiratory system compliance in mechanically ventilated covid-19 patients. Crit Care. 2021;25:199. 34108029 10.1186/s13054-021-03518-4 PMC8188162

[23] Grasselli G Tonetti T Protti A; collaborators. Pathophysiology of COVID-19-associated acute respiratory distress syndrome: a multicentre prospective observational study. Lancet Respir Med. 2020;8:1201–8. 32861276 10.1016/S2213-2600(20)30370-2 PMC7834127

[24] Zhou R Wang HT Gu W. Efficacy of high-flow nasal cannula versus conventional oxygen therapy in obese patients during the perioperative period: a systematic review and meta-analysis. Can Respir J. 2022;2022:4415313. 36247078 10.1155/2022/4415313 PMC9553645

[25] Zhu C Yao JW An LX Bai Y-F Li W-J. Effects of intraoperative individualized PEEP on postoperative atelectasis in obese patients: study protocol for a prospective randomized controlled trial. Trials. 2020;21:618. 32631414 10.1186/s13063-020-04565-y PMC7338115

[26] Abplanalp LA Ionescu F Calvo-Ayala E Yu L Nair GB. Static respiratory system compliance as a predictor of extubation failure in patients with acute respiratory failure. Lung. 2023;201:309–14. 37300706 10.1007/s00408-023-00625-7 PMC10257168

[27] Mitzner W. Mechanics of the lung in the 20th century. Compr Physiol. 2011;1:2009–27. 23733695 10.1002/cphy.c100067 PMC4562454

[28] Liu Y Ma W Liu J. Applications of airway ultrasound for endotracheal intubation in pediatric patients: a systematic review. J Clin Med. 2023;12:1477. 36836012 10.3390/jcm12041477 PMC9961112

[29] Fuller BM Page D Stephens RJ. Pulmonary mechanics and mortality in mechanically ventilated patients without acute respiratory distress syndrome: a cohort study. Shock. 2018;49:311–6. 28846571 10.1097/SHK.0000000000000977 PMC5809252

[30] Kang W Clark AR Tawhai MH. Gravity outweighs the contribution of structure to passive ventilation-perfusion matching in the supine adult human lung. J Appl Physiol (1985). 2018;124:23–33. 29051337 10.1152/japplphysiol.00791.2016 PMC5866448

[31] Thompson BT Chambers RC Liu KD. Acute respiratory distress syndrome. N Engl J Med. 2017;377:562–72. 28792873 10.1056/NEJMra1608077

[32] Ranieri VM Rubenfeld GD Thompson BT; ARDS Definition Task Force. Acute respiratory distress syndrome: the Berlin definition. JAMA. 2012;307:2526–33. 22797452 10.1001/jama.2012.5669

